# P-1470. Single-dose Aminoglycosides versus Carbapenems for the Treatment of Uncomplicated Urinary Tract Infections caused by Extended-spectrum Beta-lactamase-phenotype *Enterobacterales*

**DOI:** 10.1093/ofid/ofae631.1640

**Published:** 2025-01-29

**Authors:** Anna DeFrank, Michael Bosco, Veronica B Zafonte, Shahidul Islam, Diane Johnson

**Affiliations:** NYU Langone Hospital-Long Island, Patchogue, New York; NYU Langone Hospital - Long Island, Mineola, New York; NYU Langone Hospital – Long Island, Mineola, New York; Northwell Health, New Hyde Park, New York; NYU Langone - Long Island, Mineola, New York

## Abstract

**Background:**

Carbapenems are often used for treatment of infections caused by extended-spectrum β-lactamase-producing (ESBL) *Enterobacterales*. Over utilization has led to carbapenem resistance and these infections can be difficult to treat. Limiting unnecessary carbapenem exposure has been shown to decrease the risk of carbapenem resistance and a 2023 Infectious Disease Society of America guidance document recommends non-carbapenem alternatives for the treatment of uncomplicated urinary tract infections (uUTI). A single dose of an aminoglycoside (AG) can be considered, however clinical trial data is lacking, especially for those with infections caused by drug-resistant pathogens.

**Figure:**
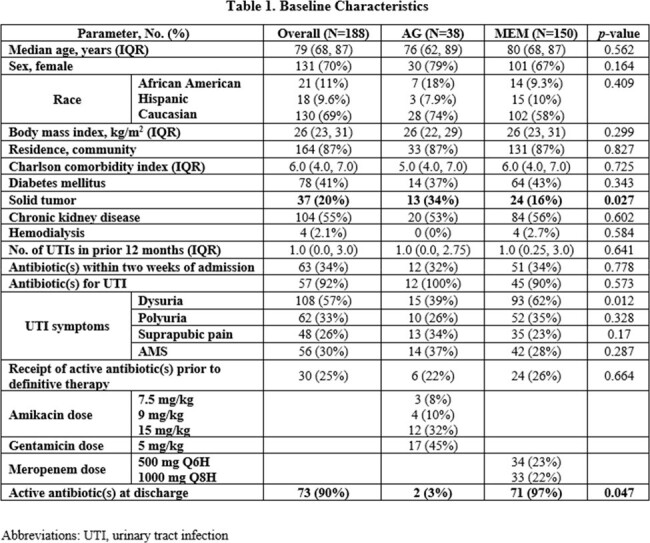

**Methods:**

A retrospective, multi-center, cohort study over 60-months evaluated hospitalized patients within the NYU Langone Health System ≥18 years of age who received a single dose of an AG for the treatment of an uUTI caused by an AG-susceptible, ESBL-phenotype (defined as ceftriaxone MIC ≥2 mcg/mL) gram-negative bacilli (GNB). Patients who received MEM for ≥72 hours were the active comparator. Clinical success, a composite of the following, was assessed at Day 7 (±2 days) post-therapy or at discharge, whichever occurred sooner: clinical cure, defined as complete resolution or significant improvement of baseline uUTI signs/symptoms; microbiological response, defined as reduction in baseline uropathogen to < 10^3^ CFU/mL. To demonstrate non-inferiority, 352 patients were needed to achieve 90% power.

**Figure:**
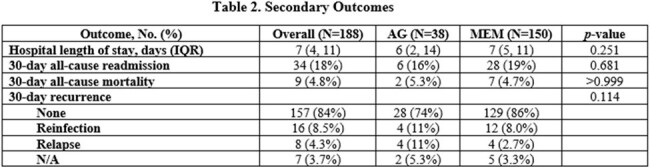

**Results:**

Baseline characteristics can be found in Table 1. Clinical success occurred in 97% (N=37) vs. 86% (N=129) of patients treated with an AG vs. MEM, respectively (p=0.052). Non-inferiority was met with a 0.11 proportion difference in clinical success (90% confidence interval [-0.011, 0.25]) within the predefined non-inferiority limit. No statistically significant differences in secondary outcomes were observed (Table 2). Diarrhea was more common in those treated with MEM (p=0.049; Table 3).

**Figure:**
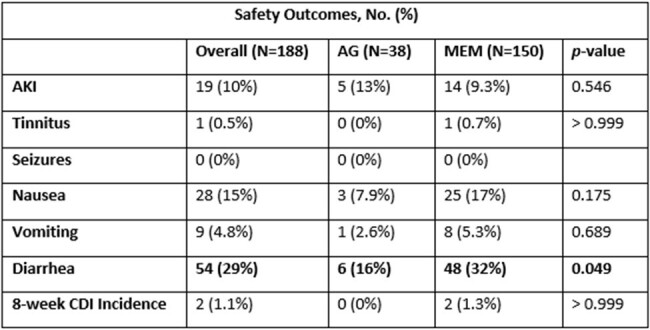

**Conclusion:**

Single dose AG demonstrated comparable clinical success and recurrence rates to MEM for the treatment of uUTI caused by ESBL-producing GNB. The study was underpowered, increasing the chance for type II error. A future prospective study is warranted to justify preliminary findings.

**Disclosures:**

**Michael Bosco, PharmD, BCIDP, AAHIVP**, Shionogi Inc: Advisor/Consultant|Shionogi Inc: Grant/Research Support

